# Marine Ostracod Provinciality in the Late Ordovician of Palaeocontinental Laurentia and Its Environmental and Geographical Expression

**DOI:** 10.1371/journal.pone.0041682

**Published:** 2012-08-10

**Authors:** Mohibullah Mohibullah, Mark Williams, Thijs R. A. Vandenbroucke, Koen Sabbe, Jan A. Zalasiewicz

**Affiliations:** 1 Department of Geology, University of Leicester, Leicester, United Kingdom; 2 Department of Geology, University of Balochistan, Quetta, Pakistan; 3 Géosystèmes, Université Lille 1, Lille, France; 4 Protistology and Aquatic Ecology, Department of Biology, Ghent University, Ghent, Belgium; Raymond M. Alf Museum of Paleontology, United States of America

## Abstract

**Background:**

We examine the environmental, climatic and geographical controls on tropical ostracod distribution in the marine Ordovician of North America.

**Methodology/Principal Findings:**

Analysis of the inter-regional distribution patterns of Ordovician Laurentian ostracods, focussing particularly on the diverse Late Ordovician Sandbian (ca 461 to 456 Ma) faunas, demonstrates strong endemicity at the species-level. Local endemism is very pronounced, ranging from 25% (e.g. Foxe basin) to 75% (e.g. Michigan basin) in each basin, a pattern that is also reflected in other benthic faunas such as brachiopods. Multivariate (ordination) analyses of the ostracod faunas allow demarcation of a Midcontinent Province and a southern Marginal Province in Laurentia. While these are most clearly differentiated at the stratigraphical level of the *bicornis* graptolite biozone, analyses of the entire dataset suggest that these provinces remain distinct throughout the Sandbian interval. Differences in species composition between the provinces appear to have been controlled by changes in physical parameters (e.g. temperature and salinity) related to water depth and latitude and a possible regional geographic barrier, and these differences persist into the Katian and possibly the Hirnantian. Local environmental parameters, perhaps operating at the microhabitat scale, may have been significant in driving local speciation events from ancestor species in each region.

**Conclusions/Significance:**

Our work establishes a refined methodology for assessing marine benthic arthropod micro-benthos provinciality for the Early Palaeozoic.

## Introduction

Ostracods are small bivalved crustaceans with a fossil record extending back to the Cambrian [1]. They are a diverse class of aquatic crustaceans [2], have a well-preserved fossil record [3], and are known from more than 65,000 living and extinct species [4]. Ostracods have adopted both benthic and pelagic lifestyles [4], [5], but most ostracods in the fossil record are benthic: the weakly calcified shells of pelagic forms are not frequently preserved [3]. The earliest ostracods occupied shelf marine benthic environments during the Ordovician [6]–[9]. Later, they colonised pelagic environments during the Silurian [10] and radiated into non-marine aquatic environments during the Carboniferous [11], [12]. Ordovician benthic ostracod distribution patterns have been used to identify biogeographical provinces (e.g., [13]–[15], to establish facies-dependent patterns (e.g., [13] and to track the relative movement of palaeocontinents [16]. As well as palaeogeographical controls on the distribution of benthic ostracods, environmental effects of temperature, substrate, food-supply and water depth are also influential (e.g., [13], [17]–[21]. Discrete latitudinal (climatically) controlled biotopes have been identified in Cenozoic fossil ostracod faunas [19], [21].

In this paper we evaluate the distributional patterns of the Ordovician ostracods of palaeocontinental Laurentia [22], focussing particularly on the faunas of Sandbian age as these are amongst the most widely studied and best known of all Ordovician ostracod assemblages (e.g., [17], [20], [23]–[27]. Laurentian Ordovician ostracods spanned a palaeolatitudinal range from 13° N to 25° S, which encompassed tropical and sub-tropical climate zones [28], [29]; they occupied a range of marine environments from peri-tidal to deep shelf, and they occur in both carbonate and clastic sedimentary deposits. Therefore, potential latitudinal and depth-related changes in temperature, substrate, productivity, oxygenation-level and salinity may be expressed in the different spatial ranges of individual taxa and ostracod biotopes. Although such patterns have previously been discerned from evaluation of ostracod presence-absence data for individual formations (e.g., [17], [20], this is the first attempt to integrate data for the whole Laurentian palaeocontinent for specific time intervals using multivariate statistical techniques.

## Results

Unlike fossil Ordovician plankton, whose distribution patterns can be evaluated from global datasets (e.g., [28]–[31], ostracods possessed no pelagic stage in their lifecycle and their primary distribution at the inter-continental scale was therefore largely controlled by geography (e.g., [13], [16]. Nevertheless, distribution patterns analysed on a continent-by-continent basis may still reflect latitudinal or environmental signatures. The research methodology used here is based on multivariate statistical assessment of presence-absence data for Laurentian species from well-defined time intervals within the Sandbian, specifically the *gracilis* and *bicornis* graptolite biozones [32]. The ‘time slab’ approach is a common method used to deal with large fossil datasets for environmental reconstruction [33], [34] and has recently been used for Late Ordovician zooplankton of Sandbian age [28]–[30].

### Ostracod Database

A Sandbian dataset (for stratigraphical definition see following section) comprising 13 regions with 229 ostracod species from 88 genera was compiled from published literature (Table S1, Appendix S1). Taxonomic filtering of the original literature data was essential to minimise error as species nomenclature has evolved over the 80 years since the first descriptions of North American Ordovician ostracods (e.g., [17], [20], [35]–[47]. Taxa described in open nomenclature or identified as “cf." and “?" were examined and only those which closely resembled their holotype were included. Taxa described as “aff." were excluded. And, taxa only identified to genus level were also excluded in order to avoid ‘noise’ in the analysis. All of the species are weighted from 1 to 4 using the following criteria, with 4 being the most reliable: 4, morphologically distinctive (lobation, marginal structures etc.), well described, stable nomenclatorial history (e.g., *Monoceratella teres* Teichert, 1937 [48]; 3, most recent descriptions taxonomically sound, some history of misidentification (e.g., *Eoaquapulex socialis* (Levinson, 1961) [49]; 2, some history of misidentification and described in open nomenclature (e.g., *Krausella*? *spinosa* (Harris, 1957) [23]; and 1, simple morphology (i.e. carapace with few or no diagnostic characters, or morphological variation poorly defined or poorly described, long history of open nomenclature (e.g., *Eurychilina*? aff. *Chilobolbina hyposulcata* sensu Kraft, 1962 [26]. Most taxa fell into categories 4 and 1. Taxa with a weighting of ‘1’ were excluded from the analysis. In our assessment of the ostracod assemblage dataset we have identified the primary sedimentary setting, taphonomy, lithology, palaeolatitude and sampling points (summarised in Table S1).

### Time Slab Definition

**Figure 1:**
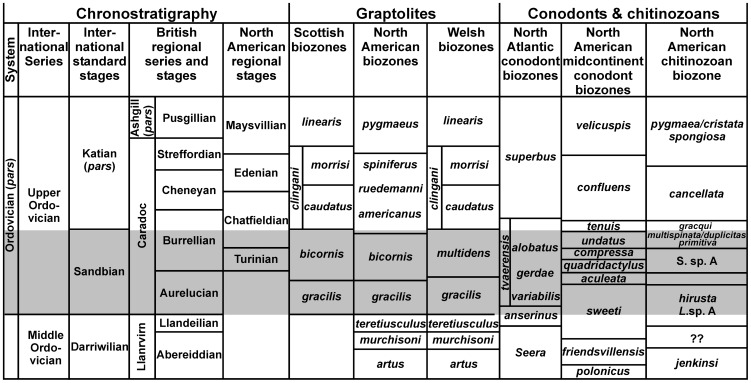
‘Sandbian time slab’ (shaded). Between the first appearance of *N. gracilis* and the beginning of the *D. clingani* graptolite Biozone. Graptolite ranges follow [82]–[86]. The conodont data are from [50] and chitinozoans are from [87]. The correlation between the graptolite, conodont, and chitinozoan biozones follows [63].

**Figure 2:**
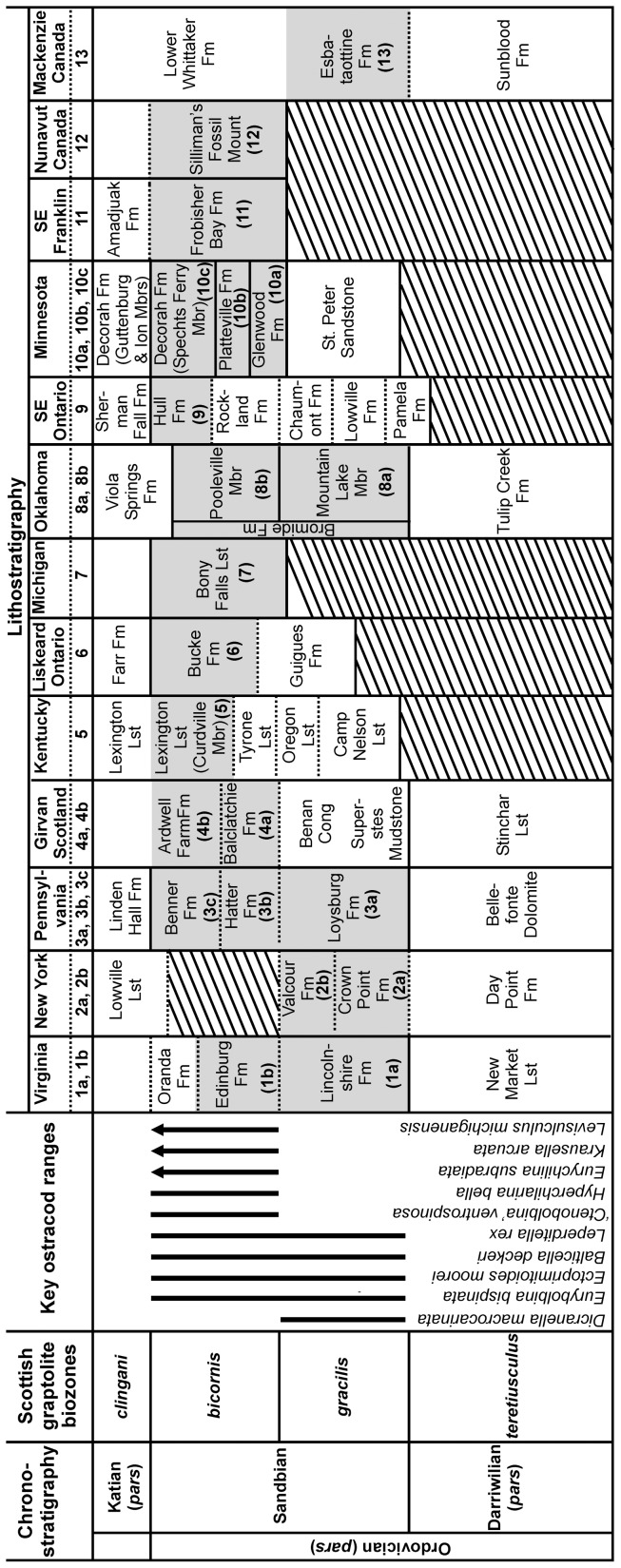
Ordovician chronostratigraphy and lithostratigraphy for North America, Canada, and the Girvan district, southwest Scotland. The North American stratigraphy follows [88], Canadian stratigraphy follows [89] and Girvan is based on [46], [90]. The Sandbian sections from which ostracods are documented and included in this study are highlighted grey.

**Figure 3:**
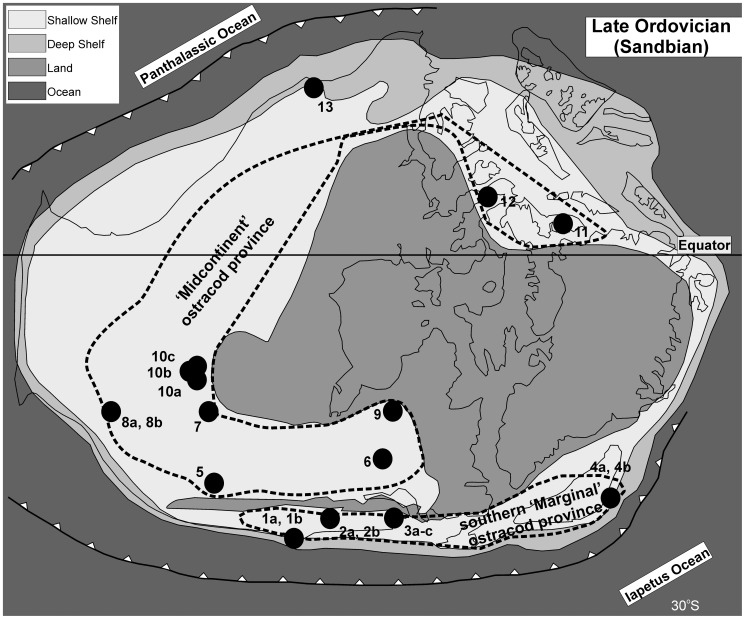
Late Ordovician (Sandbian) palaeogeography of Laurentia, the distribution of ostracod-bearing localities, and the two faunal provinces (map slightly modified after [**22**]).

The Sandbian Stage of the Upper Ordovician has been selected as a time slab for this study. It represents an interval of rock deposited from approximately 461 to 456 million years ago [32] and is well defined by the first appearance of the graptolite *Nemagraptus gracilis*. The graptolite *Ensigraptus caudatus* defines the base of the succeeding *clingani* Biozone and of the Katian Stage (Figure 1). The Sandbian is thought to represent the onset of a cooling Ordovician climate, but there is no evidence for significant climate fluctuation within the interval itself [28]–[30]. Within the Sandbian interval some 229 ostracod taxa are described (from 13 regions; Figure 2), of which 56 taxa are specifically limited to the *gracilis* Biozone (ca 3 million years duration) and 117 taxa to the *bicornis* Biozone (ca 2 million years duration; see [32] for chronology). We have analysed the distribution patterns of ostracods for the *gracilis* Biozone (5 regions; samples 1a, 2a, 2b, 3a, 8a and 13 on Table S1 and Figures 2, 3) and the *bicornis* Biozone (11 regions; samples 1b, 3b, 3c, 4a, 4b, 5–7, 8b, 9, 10a–10c, 11 and 12 on Table S1 and Figures 2, 3). Analysis of the total (Sandbian) fauna (13 regions) was also undertaken.

Graptolites are the main biostratigraphic markers used for correlating our chosen rock successions together with the coeval conodont biozones [50]. Here the upper *Pygodus anserinus* and lower *Amorphognathus tvaerensis* (*Prioniodus variabilis* Subzone) conodont biozones are considered equivalent to the *gracilis* graptolite Biozone, and the upper *A. tvaerensis* Biozone (*Prioniodus gerdae* and *Prioniodus alobatus* subzones) as equivalent to the *bicornis* graptolite Biozone [50], [51]. In some cases we have also used shelly fossils (brachiopods, trilobites and ostracods) and chitinozoans for stratigraphic correlation [47].

### Geographical spread of data

Laurentia was selected for analysis because it yields one of the most diverse and geographically widespread ostracod faunas from the Late Ordovician (Sandbian) and because it includes a broad latitudinal range (greater than 35°; Figure 3) and a wide range of palaeoenvironments [14]. We have also compared the Sandbian ostracod dataset from Laurentia with those of Avalonia (for the *gracilis* Biozone time slab) to show the relevant influence of inter-continental versus intra-continental environmental and geographical effects.

### Ordination analysis

Ordination is a tool that allows the representation of complex multivariate datasets in simple diagrams in which the axes represent the main gradients in species composition in the original dataset. These ordination axes thus represent environmental gradients (ideally the gradient of an environmental variable, but mostly a combination of several variables) which drive the gradient in species composition. In ordination diagrams, samples are ordered with respect to one another on the basis of their species composition (occurrence in the sample set) [52]. The samples that show more taxonomic resemblance are placed more closely to each other, whereas samples that show greater difference are placed apart. As a preliminary ‘Detrended Correspondence Analysis’ (DCA) using detrending by segments revealed a strong turnover in species composition between the samples in all datasets (length of gradient >6 SD, cf. Jongman et al. 1995), we used the unimodal indirect ordination method Correspondence Analysis (CA) for our analyses with the software package CANOCO for Windows 4.5 [53]. Four datasets were analyzed. We first performed a test to assess the strength of the inter-continental geographical effect on the distribution of ostracods, selecting the early Sandbian *gracilis* Biozone interval (ca 3 million years duration from 461 Ma), with five localities from Avalonia and six localities from Laurentia. We then analysed the Laurentian dataset for three time intervals, the *gracilis* Biozone (ca 3 million years), the *bicornis* Biozone (ca 2 million years), and the entire Sandbian (ca 5 million years). In order to test whether a significant stratigraphical or latitudinal/geographic signal was present in the entire Sandbian dataset, we used the direct equivalent of CA, viz. Canonical Correspondence Analyses (CCA) with stratigraphy (dummy variables for *gracilis* and *bicornis*) and palaeolatitude (absolute values of degrees palaeolatitude) as the only variables respectively. Significance was tested using Monte Carlo permutation tests (4999 unrestricted permutations, p<0,001).

## Discussion

### Inter-continental geographical analysis

For much of the Ordovician, Laurentia was separated by the Iapetus Ocean from the palaeocontinents of Baltica and Avalonia, though this ocean narrowed by the Late Ordovician [54], [55]. During the Sandbian no species are common between Laurentia and Avalonia and only a few genera are common: *Eridoconcha* during the early Sandbian (*gracilis* Biozone), and *Ceratopsis* and *Easchmidtella* during the late Sandbian (*bicornis* Biozone; [20], [43], [56]. Our results thus support the suggestion that palaeogeography exerted the strongest control over the global distribution of Ordovician ostracods (e.g. [16]. Evidently, as there are no species in common, CA analysis for the early Sandbian (*gracilis* graptolite Biozone) shows Avalonian and Laurentian localities as two discrete clusters of samples (data not shown). Ostracod fauna from the early Katian (*clingani* Biozone) of Avalonia are sparse, only six species being documented and none of these are common to Laurentia [56]. However, by the mid-late Katian and while the Iapetus Ocean was closing the Avalonian fauna became more similar to that of Laurentia at the generic level [16] and by the late Katian included the earliest common species [57].

### Analysis of the Sandbian Laurentian dataset

**Figure 4:**
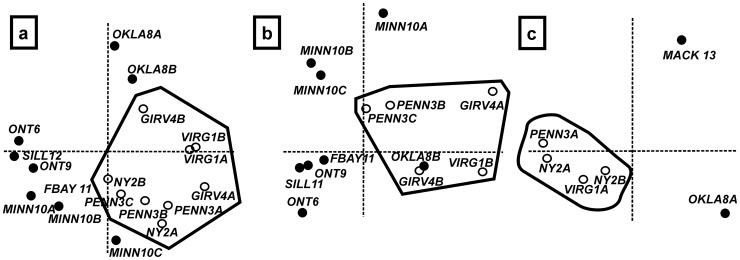
Correspondence Analyses (CA) of the (a) entire Sandbian, (b) late Sandbian (*bicornis* time slab) and (c) early Sandbian (*gracilis* time slab). Midcontinent Province localities are shown as filled circles, southern Marginal Province localities as empty circles. Grey polygons indicate the southern Marginal Province localities. For sample labels, see Table S1 and Figure 2.

A preliminary CA analysis (not shown) identified the samples from Kentucky (5), Michigan (7) and Mackenzie (13) as outliers. These three samples are characterized by the highest percentages (>65%) of endemic species in the whole dataset, and were therefore omitted from further analyses. CA analysis of the entire Sandbian (*gracilis* and *bicornis* biozones) ostracod fauna revealed a clear latitudinal signal, with all southern marginal localities lying on the right side of the first CA axis, and most midcontinent localities on the left (Figure 4a); this relation with latitude was highly significant (CCA, p<0.001). Thus, discrete Midcontinent and Marginal ostracod provinces can be recognised. The more or less intermediate position of Oklahoma is not surprising, as this was an aulacogen basin [58] that straddled the shelf to basin and therefore contained elements that are both midcontinent and marginal. No significant stratigraphical signal was present (CCA, p>0.05). Indeed, samples from regions for which both *gracilis* and *bicornis* materials were available (Virginia, Pennsylvania and Oklahoma) consistently cluster together on the basis of region, not stratigraphy (Figure 4a).

### Analysis of the bicornis biozone Laurentian dataset

**Figure 5:**
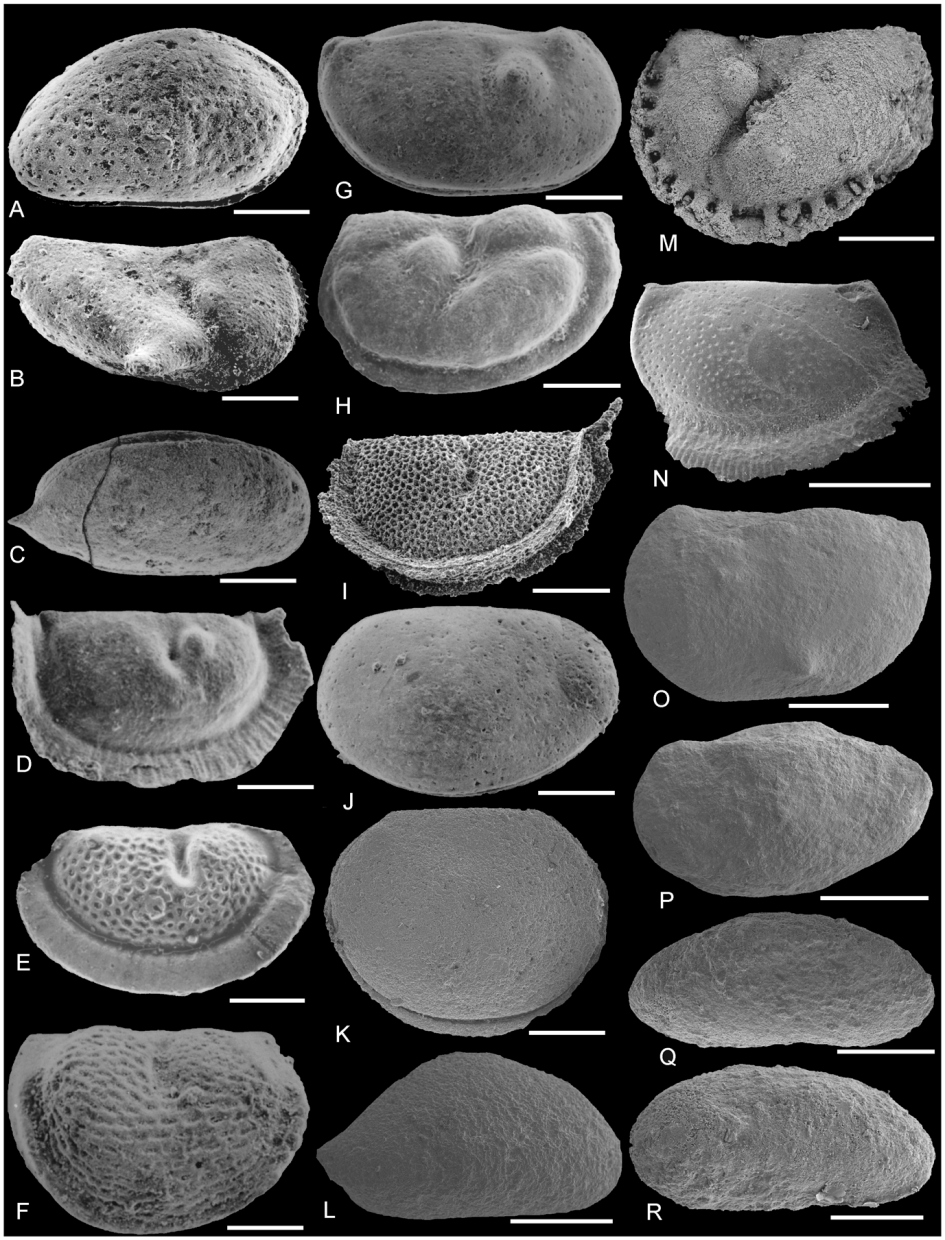
Late Ordovician ostracods of the Midcontinent and Marginal provinces and widespread (pandemic-Laurentian) assemblages of palaeocontinental Laurentia. (A–C) Sandbian Midcontinent Province assemblage; (D–L) Sandbian ostracod assemblage widespread in both the Midcontinent and Marginal provinces; (M) Sandbian Marginal Province assemblage; (N–R) Katian Marginal Province assemblage. (A) NMH UK OS13634 *Punctaparchites rugosus* (Jones, 1858) [91], carapace, right lateral view. (B) NMH UK OS13479 *Winchellatia longispina* Kay, 1940 [37], tecnomorphic right valve, lateral view. (C) MCZ 4646 *Krausella calvini* (Kay, 1940) [37], carapace right lateral view. (D) NMH UK OS13509 *Eurychilina indivisa* Levinson, 1961 [49], juvenile tecnomorphic right valve, lateral view. (E) NHM UK OS I13216 *Eurychilina reticulata* Ulrich, 1889 [92], heteromorphic right valve, lateral view. (F) NMH UK OS13535 *Hallatia labiosa* (Ulrich, 1894) [35] tecnomorphic right valve, lateral view. (G) NMH UK OS13617 *Balticella deckeri* (Harris, 1931) [38], carapace, right lateral view (H) MCZ 4599b *Eohollina depressa* (Kay, 1940) [37], tecnomorphic carapace, right lateral view (I) NMH OS13538 *Eurybolbina bispinata* (Harris, 1957) [23], juvenile tecnomorphic left valve, lateral view. (J) NMH UK OS13526 *Eoaquapulex socialis* (Levinson, 1961) [49] tecnomorphic left valve, lateral view. (K) BGS GSE 15387 *Baltonotella parsispinosa* (Kraft, 1962) [26], carapace, left valve, lateral view (L) BGS GSE 15385 *Krausella variata* Kraft, 1962 [26], right valve, lateral view. (M) BGS GSE 15384 ‘*Ctenobolbina*’ *ventrospinosa* Kraft, 1962 [26], heteromorphic, left valve, lateral view. (N) BGS 16E1961 *Oepikella tunnicliffi* Williams & Floyd, 2000 [93], heteromorphic right valve, lateral view. (O) MPA49672, *Balticella* sp., carapace, left lateral view. (P) BGS GSE15354 *Steusloffina cuneata* (Steusloff, 1895) [94], carapace, left lateral view. (Q) BGS GSE15360, *Longiscula* cf. *perfecta* Meidla, 1993 [95], carapace, left lateral view. (R) BGS GSE15365, *Longiscula* cf. *tersa* (Neckaja, 1966) [96], carapace, right lateral view. Figures (A–D, F–H, J) are from the Bromide Formation of Oklahoma; (E) is from St. Paul's Minnesota; (I) is from the Edinburg Formation of Virginia (K–M) are from the Ardwell Farm Formation, Girvan district, Scotland; (N–R) are from the Craighead Limestone Formation, Girvan district, Scotland. Scale bar (A) 210 µm; (B) 250 µm; (C) 294 µm; (D) 338 µm; (E) 320 µm; (F) 193 µm; (G) 205 µm; (H); 346 µm; (I) 346 µm; (J) 545 µm; (K–M, O, P) 500 µm; (N) 1000 µm; (Q, R) 200 µm. Repositories for specimens are: NHM, Natural History Museum, London; MCZ Museum of Comparative Zoology, Harvard University; BGS GSE, British Geological Survey, Keyworth, Nottingham.

As in the entire Sandbian dataset analysis (see paragraph above), two ostracod provinces can be distinguished for the *bicornis* Biozone time slab (Figure 4b). These are based on 173 species from 11 regions extending from Arctic Canada to Oklahoma (1b, 3b–c, 4a–b, 5, 6, 7, 8b, 9, 10a–c, 11 & 12 on Figures 2, 3; Table S1). The ostracod fauna at the geographical margins of Laurentia (1b, 3b–c, 4a–b on Figures 2, 3) shows considerable taxonomic difference from the midcontinent Laurentian fauna (Minnesota, Michigan, Kentucky, Ontario, Franklin District; 5, 6, 7, 9, 10a–c, 11 & 12 on Figures 2, 3). Oklahoma (8b on Figures 2, 3) represents the only midcontinent locality showing strong similarities with the marginal Laurentian assemblages (cf. 3.2). The Midcontinent Province comprises 48 species that are cosmopolitan across this region, of which 28 are exclusive to this province (e.g. *Krausella calvini*, *Winchellatia longispina*, *Punctaparchites rugosus*, *Phelobythocypris cylindrica*, *Saccaletia buckensis*, *Tetradella ulrichi*, *Tetradella ellipsilira*, *Dicranella bicornis*, *Pseudulrichia simplex* (and see Appendix S2; Figure 5). The Midcontinent Province also contains 88 species which have occurrences limited to a single depositional basin, and are thus endemic at a local level. The southern Marginal Province comprises 24 species that are cosmopolitan across this region, of which four are exclusive to this region (*Eurychilina strasburgensis, Shenandoia acuminulata*, ‘*Ctenobolbina’ ventrospinosa* and *Platybolbina punctata*). Within this province 33 species have occurrences restricted to a single depositional basin and are thus endemic at a local level. Oklahoma (Bromide Formation) shares ten species with the southern Marginal Province and nine species with the Midcontinent Province.

### Analysis of the gracilis biozone Laurentian dataset

The ostracod fauna from the *gracilis* Biozone of Laurentia includes materials from Virginia, New York, Pennsylvania, the Mackenzie District (Canada), and Oklahoma (1a, 2a–2b, 3a, 8a & 13 on Figures 2, 3). Out of 111 species 88 species are endemic to a single basin and only a few species (23) are common to several localities (Appendix S3). CA analysis of this limited *gracilis* dataset suggests the presence of a latitudinal signal (Figure 4c), but more data are needed to confirm this.

### ‘Midcontinent’ and ‘Marginal’ ostracod provinces explored

The midcontinent was characterised by carbonate platforms, whilst the margins were typically ramp settings characterised by carbonates and mudstones with a broader range of facies from peri-tidal to outer ramp settings. Studies of bryozoans, corals, conodonts and trilobites [59]–[63] have distinguished discrete Laurentian provinces in the Ordovician, largely controlled by depth-related lithofacies, climate and sea level change. All the above mentioned faunal groups broadly show the same distribution pattern as the ostracods. Each displays distinct marginal faunas which differentiate them from the midcontinent faunas [60]. The ostracod distribution patterns can be closely correlated with those for brachiopods. The Middle and Late Ordovician brachiopod faunas show shallow benthic brachiopod assemblages in midcontinent Laurentia, whereas a broader range of brachiopod biofacies were developed in both eastern and western margins of the palaeocontinent [64].

The factors that may control the two ostracod provinces are those associated with geography, water depth (e.g. temperature, salinity), latitude (climatic), and substrate.

### Geography

The Palaeozoic geography of Laurentia has been reviewed and discussed in detail by piecing together information from palaeomagnetic studies and faunal distribution patterns [22]. They demonstrated that for most of the Ordovician the central part of the Laurentian craton was stable whereas the margins were tectonically active. Epeiric seas also repeatedly flooded the Laurentian craton that resulted in thick successions of Ordovician carbonate platforms [22], [65]. The distribution patterns of most of the fossil groups that show distinct assemblages in the marginal and midcontinent regions are widely regarded as differences between depositional environments [22], [61], [62], [66]. However, a peninsula land mass existed between the different regions and may, at least, have partly separated the southern margin from the midcontinent area (see [22]; figures 8, 11). This landmass might have formed a geographic barrier for exchange of ostracods and other benthic faunal groups.

### Substrate

Seabed substrate is recognised as an important factor in the distribution of Ordovician ostracods at a continental scale [13]. For example, the carbonate facies of Baltoscandia are dominated by palaeocope-rich assemblages, whereas those from the Armorican Massif are dominantly mudstone lithofacies with binodicope-rich assemblages [13]. The dominance of binodicopes is also noticed in the Ordovician mudstones of Saudi Arabia and southern Britain [13]. The Laurentian dataset includes ostracods sourced from both clastic, carbonate and mixed carbonate-clastic lithologies (Table S1). Palaeocopes are the dominant group (see Appendix S1) in both the carbonates and mudstones. For example, the high diversity fauna of the shale unit of the Bucke Formation of Ontario is dominated by palaeocopes [27] as are the limestone facies of the Hatter and Benner formations of Pennsylvania [25]. Thus, the dominance at mid to high palaeolatitude (Armorican Massif, Saudi Arabia and southern Britain) by binodicopes, whilst low palaeolatitudes (Baltoscandia, Laurentia) are dominated by palaeocopes may also be related to latitudinal temperature change and not to substrate control alone. Therefore, while substrate may have affected ostracods at the very local level, perhaps indicated by the high degree of species-level endemicity in each basin, it is not clearly expressed in the distribution patterns of binodicope-rich and palaeocope-rich ostracod assemblages at a provincial scale in Laurentia.

### Water depth

In previous studies of Late Ordovician ostracods water depth has been considered to have a strong influence on the distribution of ostracods [17], [20], [67]. Thus, the two biofacies in the lower Esbataottine Formation of the Mackenzie district, Canada, are interpreted as a deeper platform biofacies and a shallow shelf biofacies that also has some elements extending into deeper shelf facies [17]. Similarly, a peri-tidal ostracod biofacies and an open-shelf biofacies are recognised in the carbonate ramp setting of the Bromide Formation of Oklahoma [20]. However, the shallow and deep shelf assemblages of the lower Esbataottine Formation are of questionable significance when the ostracod fauna is considered on the continental scale. Most of the supposed deeper platform taxa of the lower Esbataottine Formation are found in shallow marine facies elsewhere. These include species of the genera *Eohollina*, *Platyrhomboides*, *Dicranella*, *Cryptophyllus*, *Winchellatia*, *Baltonotella*, *Tetradella* and *Euprimitia*. The former six of these are found in shallow to deep shelf facies of the Bromide Formation of Oklahoma, whereas species of *Euprimitia* are found in shallow shelf facies of the Crown Point Formation of New York and species of *Tetradella* are present in the shallow shelf facies of the Hull Formation of Ontario [20], [25], [36]. The water depth assemblages of the Esbataottine Formation were based on generic-level assessments, which may be, at best, diagnostic only locally and cannot be traced on the pan-Laurentian scale [17]. The Bromide Formation's shallow and deep shelf assemblages have only a few species that are widespread elsewhere. Some of the diagnostic deep shelf taxa of the Bromide Formation of Oklahoma such as *Baltonotella parsispinosa* are also present in the shallow shelf facies of the Crown Point Formation of New York and deep shelf facies of the Edinburg Formation of Virginia [20], [25], [26]. Similarly, *Eurybolbina bispinata* that occurs only in the deep shelf of the Bromide Formation is also present in the shallow to deep shelf facies of the lower Esbataottine Formation of the Mackenzie District and deep shelf facies of the Lincolnshire and Edinburg formations of Virginia [17], [20], [26]. Nevertheless, analysis of the pan-Laurentian dataset does identify some diagnostic species that characterize shallow and deep shelf facies of the Bromide Formation in similar settings elsewhere. *Leperditella rex* in peri-tidal and innermost shelf facies of the Bromide Formation is also present only in the peri-tidal facies of the Hatter and Benner formations of Pennsylvania and the inner shelf facies of the Bucke Formation of Ontario [20], [25], [27]. The distribution patterns of the Laurentian ostracod fauna may therefore reflect some depth-related physical parameters between the ‘Marginal’ and the ‘Midcontinent’ provinces.

### Latitude

Palaeonvironmental change associated with palaeolatitude exerted a strong influence on the distribution of fossil marine organisms [21], [28], [29]. The distribution patterns of zooplankton have already been shown to reflect climate zones in the Palaeozoic, Mesozoic and Cenozoic [21], [28], [29], [68]–[70]. The distribution of Cenozoic benthic ostracods have also been demonstrated to be influenced by latitude [21], [71]. The same pattern of latitudinal-restricted assemblages may also be reflected in Ordovician ostracods as the Laurentian ostracod provinces identified here are restricted to relatively narrow latitudinal ranges. The southern Marginal Province localities are restricted to latitudes 21–25°S whereas the Midcontinent Province is confined to 17°S-5°N and both of these are characterized by species with a restricted latitudinal range (Figure 2). These include *Platybolbina punctata*, ‘*Ctenobolbina’ ventrospinosa*, *Shenandoia acuminulata* and *Eurychilina strasburgensis* from the Marginal Province. Also, some 28 species (*Hyperchilarina bella*, *Tetradella ellipsira*, *T. Ulrichi* etc.) are restricted only to the Midcontinent Province (Appendix S2). This suggests that latitudinal temperature variation may have been a factor in the distribution of the Laurentian ostracod fauna, particularly as this is also reflected in the boundary between the tropical and subtropical climate zone determined at about 22°S from the analysis of zooplankton [29].

### Trans-Iapetus ostracod connections between Laurentia and Baltica

Schallreuter and Siveter [16] demonstrated generic-links between Laurentia, Baltica and Avalonia commencing during the late Darriwilian (late Middle Ordovician). They argued that faunal similarity from the Middle to Late Ordovician was indicative of a narrowing Iapetus Ocean and the ability of some species to migrate across this ocean. By Late Ordovician times, there were early species-level links between Laurentia, Baltica and Avalonia [46], [57], [67].

The southern Marginal Province ostracod faunas of Laurentia established the earliest faunal links with the Baltic region during the early Darriwilian, as seen by the presence of species of *Rivillina* and *Laccochilina* in the Kanosh Shale of Utah [72], species of these genera being also present in approximately contemporaneous deposits of the Baltic region (see [13]. By the early Late Ordovician (*bicornis* Biozone) both the Marginal province faunas and the Midcontinent Province faunas possessed generic links with Baltica [14] and Avalonia (this study). Thereafter, the Marginal Province faunas produced the first species-level links with Baltica and Avalonia during the Late Ordovician [57], [67], [73]. Many species (*Longiscula perfecta*, *L. tersa*, *Medianella longa*, *Steusloffina cuneata* and species of *Hemiaechminoides* and *Kinnekullea*) that are restricted to the Marginal Province of Laurentia are also present in the early Katian of the Baltic region (Figure 5; [67]. Similar patterns of strong affinities between Laurentian marginal faunas across the Iapetus Ocean are also noticed in brachiopods, trilobites, conodonts and bryozoans [22], [61], [62], [74].

### Causes of intra-continental ostracod endemism in Laurentia

At species-level, endemism amongst North American Sandbian-age ostracods is pronounced, and reflects patterns that were already firmly established in earlier Dapingian and Darriwilian ostracod faunas [23], [72]. Of 229 Sandbian species documented here, only 65 species occur in more than one sedimentary basin. A few Sandbian taxa are truly pandemic to Laurentia and include *Baltonotella parsispinosa*, *Hallatia labiosa*, *Eoaquapulex socialis*, *Eurychilina subradiata*, *E. ventrosa*, *Macrocyproides trentonensis*, *Phelobythocypris cylindrica* and *Cryptophyllus oboloides* (Appendix S1). Endemism is most prominent in the successions of Oklahoma (Southern Oklahoma Aulacogen Basin), Virginia (East Shenandoah Valley Basin), the Mackenzie District (Root River Basin), Michigan and Lake Timmiskaming, Ontario, Canada. All of these areas present more than 50% endemic species that are restricted to their particular depo-centre and are not found elsewhere (Table S1). Similar striking differences at species level persist into the Katian (*clingani* graptolite Biozone interval; for which see [36], [37], [67].

The strong intra-continental endemism at the species-level in the Laurentian ostracod fauna suggests that rapid speciation was taking place from ancestor taxa in each basin. The comparative rate of speciation is different for different faunal groups [75]. In marine benthic ostracods the rate of speciation can be completed in less than 0.5 million years [76]–[78]. Speciation may have been driven by both biotic (competition) and abiotic factors such as geographic habitat, geographic isolation, climate, tectonics, and sea level change [75], [77]. Geographic isolation formed by barriers such as large deep-water bodies or islands may result in speciation from founder species as noticed across the Isthmus of Panama for ostracods [79], [80], Notwithstanding the presence of a peninsula between the Midcontinent and Marginal ostracod provinces, that may have fostered allopatric speciation, the profound endemism of ostracod faunas between individual basins in both the Midcontinent and Marginal provinces suggests environmental factors operating at the micro-habitat scale in each depositional basin may have profoundly influenced the path of ostracod evolution.

Similar patterns of strong endemism at the species-level are reported from other Ordovician fossil groups. Hansen and Holmer [81] reported a diverse brachiopod fauna from the Lower and Middle Ordovician (late-Floian to mid-Darriwilian) of Spitsbergen bearing strong generic affinities with faunas from the rest of Laurentia. However, at species-level the Spitsbergen fauna is dominated by local endemics, with only 13 from 60 species found elsewhere in North America. They also related endemism to local environmental effects influencing the evolution of taxa that had migrated from elsewhere.

### Conclusions

Analysis of the distribution patterns of Late Ordovician Laurentian ostracods demonstrates that:

1. Inter-continental geography exerts the strongest control on ostracod distribution, the faunas of Laurentia and Avalonia plotting as discrete entities for the early Sandbian, and supporting palaeogeographical reconstructions for this time interval;
2. Within Laurentia there is strong endemicity at the species-level in each depositional basin, ranging from 25% (e.g. Foxe basin) to 75% (e.g. Michigan basin), with the exception of Girvan which is an allochthonous fauna;
3. Multivariate analyses of the entire Sandbian, *gracilis* and *bicornis* time slabs allows for demarcation of Midcontinent and southern Marginal ostracod provinces;
4. Midcontinent and southern Marginal ostracod provinces appear to persist from the Sandbian into the Katian, and faunal contacts with Baltica and Avalonia are strongest with the Marginal Province, including the first species-level links, possibly reflecting greater geographical proximity and water depth tolerance of these faunas;
5. The Midcontinent and southern Marginal provinces could, in part, reflect the Tropical and Subtropical climate belts that have earlier been identified based on zooplankton distributions;
6. The strong regional endemicity of the Laurentian ostracod fauna at species-level is reflected in other faunas such as brachiopods. The strong endemicity suggests that local environmental parameters operating at the microhabitat scale may have been significant in driving local speciation events from ancestor species in each depositional basin.

## Supporting Information

Table S1 **Sandbian ostracod localities of North America, Canada and southwest Scotland.** Ostracod distribution shown by lithology, depositional setting, palaeolatitude, sampling points, taphonomy, total number of species in each Formation and basin, and the number and percentage of endemic species in each depositional basin.
(DOCX)

Appendix S1 **Entire Sandbian time slab ostracod dataset of palaeocontinental Laurentia.**
(XLSX)

Appendix S2 **Sandbian (** ***bicornis*** **) time slab ostracod dataset of palaeocontinental Laurentia.**
(XLSX)

Appendix S3 **Sandbian (** ***gracilis*** **) time slab ostracod dataset of palaeocontinental Laurentia.**
(XLSX)
