# Supplementary material for: Marine Ostracod Provinciality in the Late Ordovician of Palaeocontinental Laurentia and Its Environmental and Geographical Expression
Source: PLoS One. 2012 Aug 10;7(8):e41682. doi: 10.1371/journal.pone.0041682 (PMC3416799; doi:10.1371/journal.pone.0041682)
Supplement: Table S1 — Sandbian ostracod localities of North America, Canada and southwest Scotland. Ostracod distribution shown by lithology, depositional setting, palaeolatitude, sampling points, taphonomy, total number of species in each Formation and basin, and the number and percentage of endemic species in each depositional basin. (DOCX) [file pone.0041682.s001.docx]

| **Lithostrati-graphy** | **Samples** | **Lithofacies** | **Depositional** **environment** | **Taphonomy** | **Palaeo-latitude** | **Location** | **Basin** | **No. of sampling points** | **No. of species** | **No. & % ge of endemic species in each basin** | **References** |
| --- | --- | --- | --- | --- | --- | --- | --- | --- | --- | --- | --- |
| Lincolnshire Formation | 1a | Limestone and chert | Mid to outer ramp | Apparently autochthonous | 25^0^S | Virginia | Eastern basin Shenandoah valley basin | 15+ | 22 | Collectively 27 (57%) out of 47 species from Virginia are endemic | [26] |
| Edinburg Formation | 1b | Argillaceous limestone & calcareous shales | Outer ramp | Apparently autochthonous | 25^0^S | Virginia |  | 20+ | 43 |  | [26] |
| Crown Point Formation | 2a | Limestone | Inner ramp | Apparently autochthonous | 22^0^S | New York | Appalachian basin | 10+ | 34 | Collectively 24 out of 49 (49%) species from the Appalachian basin are endemic | [24,25] |
| Valcour Formation | 2b | Limestone & dolomite | Inner ramp | Apparently autochthonous | 22^0^S | New York |  | 5+ | 3 |  |  |
| Loysburg Formation | 3a | Limestone & dolomite | Mid ramp | Apparently autochthonous | 22^0^S | Pennsylvania |  | 5+ | 12 |  |  |
| Hatter Formation | 3b | Limestone | Peritidal | Apparently autochthonous | 22^0^S | Pennsylvania |  | 5+ | 14 |  |  |
| Benner Formation | 3c | Limestone | Peritidal | Apparently autochthonous | 22^0^S | Pennsylvania |  | 5+ | 9 |  |  |
| Lexington Limestone | 5 | Limestone | Inner ramp | Apparently autochthonous | 20^0^S | Kentucky |  | 4 | 6 |  | [45] |
| Balclatchie Formation | 4a | Mudstones | deep marine mudstones | Allochthonous | 21^0^S | Girvan, Scotland | Girvan basin | ? | 2 | 0 (Girvan fauna is completely allochthono-us, so no endemic species) | [46] |
| Ardwell Farm Formation | 4b | Mudstones | deep marine mudstones | Allochthonous | 21^0^S | Girvan, Scotland |  | 2+ | 5 |  | [46,47] |
| Bucke Formation | 6 | Limestone, dolomite and shales | Inner ramp | Apparently autochthonous | 17^0^S | Ontario, Canada | Timiskaming outlier | 20+ | 49 | 26 (53%) | [27] |
| Bony Falls Limestone | 7 | Limestone | Inner ramp | Apparently autochthonous | 13^0^S | Michigan | Michigan basin | 10+ | 12 | 9 (75%) | [41,42] |
| Bromide Formation | 8a, 8b | Limestone with few interbedded shales and sandstone | Peritidal to Mid ramp | Autochthonous | 13^0^S | Oklahoma | Oklahoma Aulacogen | 100+ | 53 | 29 (56%) | [20] |
| Hull Formation | 9 | Limestone & shales | Inner ramp | Apparently autochthonous | 13^0^S | Ontario, Canada | Ottawa basin | ? | 17 | 5 (29%) | [36] |
| Platteville Formation | 10a | Limestone | Open marine shelf | Apparently autochthonous | 11^0^S | Platteville, Minnesota | Illinois basin | ? | 18 | 16 (42%) out of 38 species from the Illinois basin are endemic | [39] |
| Spechts Ferry Member Decorah Formation | 10b | shales with few thin beds of Limestone | Open marine subtidal shelf | Apparently autochthonous | 11^0^S | Minnesota NA |  | ? | 20 |  | [40] |
| Glenwood Formation | 10c | Shales | Outer shelf | Apparently autochthonous | 10^0^S | Glenwood, Minnesota |  | ? | 5 |  | [39] |
| Silliman’s Fossil Mount | 12 | Calcareous shales & Limestone | Mid to outer ramp | Apparently autochthonous | 5^0^N | Franklin, Canada | Foxe basin | 20+ | 30 | 8 out of 32 species (25%) are endemic | [43] |
| Frobisher bay Formation | 11 | limestone with some interbedded shales | Inner ramp | Apparently autochthonous | 3^0^N | Baffin Island Nunavut, Canada |  | ? | 4 |  | [44] |
| Lower Esbataottine Formation | 13 | Limestone | Inner to mid-ramp | Apparently autochthonous | 13^0^N | Mackenzie, Canada | Root river basin | 30 | 31 | 20 (65%) | [17] |
